# Global Standards for Ophthalmology Examinations and Accreditation of ICO Exams by Society Members

**Published:** 2016

**Authors:** Fatemeh HEIDARY, Reza GHAREBAGHI

**Affiliations:** 1 Immunoregulation Research Center, Shahed University, Tehran, Iran; 2 International Virtual Ophthalmic Research Center (IVORC)

**Keywords:** Exam, International Council of Ophthalmology (ICO), Medical Education, Educational Measurement, Ophthalmology

The foremost aim of medical education is improvement of health for all human beings. This is the goal of the World Federation for Medical Education (WFME). The WFME is an international organization representing all medical doctors and teachers, as well as teaching institutions. Along with publishing its workbook, the WFME aims to implement the highest ethical and scientific standards in medical teaching and to encourage the advancement of new instructional tools, learning methods, and innovative management of medical teaching. The worldwide focus of the WFME is on the evaluation of medical trainees, with a concentration on validity, reliability, and fairness of assessment tools (1).

A growing number of publications have stressed the necessity of worldwide evaluations of medical trainees. However, despite the existence of various governmental and non-governmental institutions for certifying and evaluating ophthalmology residents, international consensus and universally accepted standards for this purpose do not yet exist. Nevertheless, as the only international council that conducts medical-specialty examinations, the International Council of Ophthalmology (ICO) investigated the curricula used for ophthalmology trainees in a number of countries. This investigation was designed and reviewed by a working party in order to establish a curriculum for residency training.

Currently, there is high global demand for qualified ophthalmologists committed to vigilance and rehabilitation of vision. This need is especially evident in developing countries that lack well-trained specialists. To meet this need, the ICO has established several examinations on topics including basic science, optics and refraction, instrument use, and clinical sciences. Any individual who passes these three-step examinations or any similar examination may attend the advanced ICO examination (2). These ICO examinations have high scientific quality and acceptability and follow the highest educational standards in ophthalmic care. However, the challenge is to secure acknowledgment of these exams by local ophthalmic societies worldwide. According to the Royal College of Physicians and Surgeons of Glasgow, candidates who pass the ICO basic science and optics and refraction exams are exempt from Glasgow’s stage one exam and those that pass the ICO clinical science exam are exempt from Glasgow’s part two exam (multiple choice questions only) (3). 

However, to date there are no uniform criteria for evaluation among ICO society members. For instance, the evaluation of residents (practical, written, and oral) is carried out every two years in Malawi, while in Germany a 30-minute oral exam is considered the final assessment exam after a training period of 60 months (4). 

Determining the measurable scale to use is the first step in resolving this problem, which by itself is considered a major challenge in creating any international policies. However, if the ICO plans to launch a uniform assessment style among its member societies worldwide, it should establish global policies both for providing clear society databases and contact details and for ensuring the commitment of those societies to the implemented roles. In other words, stakeholders should respect ICO strategies and recognize its exams as equivalent to their national exams. Societies need to appreciate that no real borders exist in the current fast-growing scientific realm. To allude to this fact, we would like to cite a 2010 report in the journal Lancet (5): “all health professionals in all countries should be educated to mobilize knowledge and to engage in critical reasoning and ethical conduct so that they are competent to participate in patient and population-centered health systems as members of locally responsive and globally connected teams.” 

In summary, we believe that the major authorities in the field of ophthalmology should implement uniform, fair, and strict universal standards for the evaluation of ophthalmology residents worldwide. Following implementation of those standards, a continuous quality control and auditing approach should be used. In addition, evaluation of each program in view of its advantages and disadvantages should be performed in parallel to promote quality and betterment of that specific program. This approach would provide us with a better and more applicable plan for the continuance of this uniform assessment style. We who have set out on this mission should appreciate this fact. Synchronous globalization in all fields of science is rapidly growing and will definitely affect medical education. Once international standards are adopted, we should respect them, although local barriers, such as market forces and resistance from powerful lobby groups, may be anticipated in advance. Possible approaches for the enforcement of the adopted standards should then be proposed, as some obstacles at the national level may still exist for young ophthalmologists who are entering practice. 
